# Supplementary material for: Learning curve and surgical outcome of robotic assisted colorectal surgery with ERAS program
Source: Sci Rep. 2022 Nov 29;12:20566. doi: 10.1038/s41598-022-24665-w (PMC9709162; doi:10.1038/s41598-022-24665-w)
Supplement: Supplementary file 1 — Supplementary Information 1. [file 41598_2022_24665_MOESM1_ESM.zip › LSC Raw data-Ñ[▒K/089/02_ñw╝╨░O▒Kñσ.pdf]

正式摘要(電子簽章：000000196618487)

W52 / W52

臺中榮民總醫院

[redacted]

- 手術日期：2020/08/11
- 手術醫師：VS 蔣鋒帆，CR 黃俋霖，CR 陳映菁，R 林志安
- 執行麻醉：VS 徐煥
- Anesthesia : GE
- Position : Supine
- Aseptic agents : Soap , Betadine Alcohol , Betadine Aqua

Diagnosis

- Pre-operative diagnosis : rectal cancer s/p laparoscopic LAR with suspected leakage
- Post-operative diagnosis : Ditto.

Surgical Indication

- anastomosis leak with septic shock, for intestinal content bypass

Operation

- Loop Ileostomy

Finding

- 1.dirty ascites and mild small intestinal distension
  2.Edematous small intestine was noted

Procedure

- 1. Patient was put on supine position, and GE anestehsia was applied.
  2. We steriled and prepare the operation field as usual.
  3. RLQ wound was opened deep into peritoneal cavity
  4. Cecum, appendix and termial ileum was localized.
  5. Small intestine was pulled out(around 10 cm proximal to terminal ileum)(serosa tear site) from RLQ wound and rod was inserted to mesentary side of ileum to support it.
  6. Small intestine was cut, and 4-0 monocryl was used for loop ileostomy suture.
  7. Loop ileostomy was patent and then the patient was sent to POR for further care.
- Estimated blood loss: minimum
- 器械數量: 正確
- 紗布數量: 正確

主任 2062F蔣鋒帆 / 醫師 2303K陳映菁

正式摘要(電子簽章：000000196618487)

W52 / W52

doctorRank:B
